# Development and Sequential Analysis of a New Multi-Agent, Anti-Acne Formulation Based on Plant-Derived Antimicrobial and Anti-Inflammatory Compounds

**DOI:** 10.3390/ijms18010175

**Published:** 2017-01-17

**Authors:** Crina Saviuc, Bianca Ciubucă, Gabriela Dincă, Coralia Bleotu, Veronica Drumea, Mariana-Carmen Chifiriuc, Marcela Popa, Gratiela Gradisteanu Pircalabioru, Luminita Marutescu, Veronica Lazăr

**Affiliations:** 1 Department of Botany-Microbiology, Research Institute of the University of Bucharest-ICUB, University of Bucharest, Faculty of Biology, SplaiulIndependenţei 91-95 Sector 5, 76201 Bucharest, Romania; crina.saviuc@yahoo.com (C.S.); ciubuca.b@gmail.com (B.C.); cbleotu@yahoo.com (C.B.); bmarcelica@yahoo.com (M.P.); gratiela87@gmail.com (G.G.P.); lumidascalu@yahoo.com (L.M.); veronica.lazar2009@gmail.com (V.L.); 2 S.C. SANIMED INTERNATIONAL IMPEX S.R.L., Sos. Bucuresti-Magurele, nr.70, Sector 5, 051434 Bucharest, Romania; 3 S.C. Biotehnos S.A., Gorunului Str. 3-5, 075100 Otopeni, Ilfov, Romania; gabi.dinca@biotehnos.com (G.D.); drume_67@yahoo.com (V.D.); 4 Ştefan S. Nicolau Institute of Virology, 285 Mihai Bravu Avenue, Sector 3, 030304 Bucharest, Romania

**Keywords:** plant-derived compounds, antimicrobial, acne, eugenol, β-pinene, eucalyptol, limonene

## Abstract

The antibacterial and anti-inflammatory potential of natural, plant-derived compounds has been reported in many studies. Emerging evidence indicates that plant-derived essential oils and/or their major compounds may represent a plausible alternative treatment for acne, a prevalent skin disorder in both adolescent and adult populations. Therefore, the purpose of this study was to develop and subsequently analyze the antimicrobial activity of a new multi-agent, synergic formulation based on plant-derived antimicrobial compounds (i.e., eugenol, β-pinene, eucalyptol, and limonene) and anti-inflammatory agents for potential use in the topical treatment of acne and other skin infections. The optimal antimicrobial combinations selected in this study were eugenol/β-pinene/salicylic acid and eugenol/β-pinene/2-phenoxyethanol/potassium sorbate. The possible mechanisms of action revealed by flow cytometry were cellular permeabilization and inhibition of efflux pumps activity induced by concentrations corresponding to sub-minimal inhibitory (sub-MIC) values. The most active antimicrobial combination represented by salycilic acid/eugenol/β-pinene/2-phenoxyethanol/potassium sorbate was included in a cream base, which demonstrated thermodynamic stability and optimum microbiological characteristics.

## 1. Introduction

Acne is a prevalent, chronic skin disorder that can sometimes evolve towards severe outcomes and affects about 85% of adolescents and 50% of adults 20 years or older [1].

Many over-the-counter acne products including gels, cleansing lotions, foams and towelettes, leave-on products, and treatment kits are available to treat mild to moderate acne or periodic breakouts. Acne products work in various ways, depending on their active ingredients (benzoyl peroxide, salicylic acid, sulfur, and α hydroxyl acids): some acne products work by killing the microorganisms causing the inflammation, while others remove excess oil from the skin or speed up the turnover of skin cells. Most of the commercially available acne products have certain drawbacks. Topical therapies, such as benzoyl peroxide, retinoids, antibiotics, and salicylic acid, may cause skin irritation, leading to a lack of patient adherence. Similarly, oral isotretinoin, which is a highly effective medication for acne, usually causes dry skin, cheilitis, and photosensitivity [2]. In addition, antimicrobial resistance is a very important disadvantage of these products. The development of resistance to antimicrobial agents used in the treatment of acne is multifactorial, a consequence of monotherapy, long-term administration, overuse, or incorrect administration in sub-inhibitory concentrations [3,4]. The resistance mechanisms activated in skin microbiota and pathogens under the selective pressure exhibited by antibiotics can be transferred to other bacterial autochthonous or allochthonous species. Resistance to current antimicrobial therapies as a result of antibiotics use has raised the need to explore new antimicrobial agents against acne. A study performed in the US in the 1990–2002 has revealed a significant decline in the use of antimicrobial drug classes (benzoyl peroxide, topical clindamycin, oral erythromycin, and tetracycline) with a shift towards non-antibiotic treatment in acne management, including topical retinoids and oral isotretinoin. However, there are increasing concerns regarding the teratogenicity of some compounds (i.e., isotretinoin), highlighting the clear need for therapeutic alternatives [5].

The antibacterial and anti-inflammatory potential of natural, plant-derived compounds has been reported in many studies. Emerging evidence shows that plant-derived essential oils and/or their major compounds may represent a plausible alternative treatment for acne [1]. In vitro and in vivo studies have revealed that essential oils mixtures, oleoresin, flavonoids, alkaloids, phenol and phenolic compounds, tannin, xanthone and xanthone derivatives, diterpene acid, phenylpropanoid glycosides, acteoside, and the bisnaphthquione derivatives are effective in the treatment of acne due to their antimicrobial and anti-inflammatory activities [6,7]. Over-the-counter acne treatments containing tea tree oil from the plant *Melaleuca alternifolia* are generally available and represent a common choice amongst patients treating themselves [8]. Thai sweet and holy basil oil rich in methyl chavicol (93.0%) and eugenol (41.5%) exhibited in vitro activity against *Propionibacterium acnes*, and proved their potential for use in suitable formulations for acne skin care [9]. Rosemarinic acid (ROA), a naturally occurring ester of caffeic acid, induces nucleoid damage with an increase in spatial division and condensation of genetic material, exhibiting antibacterial activity against many human pathogenic bacterial strains, including *P. acne*. The ROA-loaded niosome included in a gelling agent exhibited a sustained in vitro antimicrobial activity against *P. acne* and *Staphylococcus aureus* [10]. *Citrus sinensis* and *Ocimum basilicum* L. essential oil (EO) gel formulations provided good to excellent results in the treatment of acne volunteers because of their antiseptic and keratolytic activity [11]. Multi-agent therapy is common across all medical specialties and diagnoses based on the well-accepted concept that drugs used in combination can provide complementary, synergic effects, which may improve the therapeutic outcomes and thus patient compliance [12].

Therefore, the aim of this study was to develop and test the antimicrobial activity of a new multi-agent, synergic formulation based on plant-derived antimicrobial compounds (i.e., eugenol, β-pinene, eucalyptol, and limonene) and anti-inflammatory agents for its potential use in the topical treatment of acne and other skin infections.

## 2. Results and Discussion

The etiology of acne lesions is multi-factorial, involving altered lipogenesis, sebum production, hyperkeratinization, proliferation/differentiation of sebocytes, and cytokine expression. Therefore, the management of acne is a long-standing and personalized process, requiring a multi-pharmacological approach, targeting as many of the above-mentioned pathological processes as possible [13]. The purpose of this study was to evaluate the antimicrobial activity and possible mechanisms of action of a combination of plant compounds in order to propose a new topical formulation for acne management. The antimicrobial activity of the proposed formulation was assessed against Gram-positive, Gram-negative, and fungal strains. These strains have been chosen for the following reasons: (i) they are indicated by the US Pharmacopeia for antimicrobial effectiveness testing [14]; (ii) there are many cases of complications due to *P. aeruginosa* and *S. aureus* following long-term antibacterial treatment in acne patients, which are generally underestimated, since correct sampling and bacteriology is rarely performed by clinicians [15,16,17]; (iii) moreover, long-term use of antibiotics in the treatment of acne vulgaris can lead to the emergence of antimicrobial resistance both in *Propionibacterium acnes* and other bacterial species, with systemic consequences [18].

### 2.1. Antibacterial Activity Assays

The qualitative screening allowed for the classification of compounds depending on the diameter of the microbial growth inhibition zone around the spotted area. All tested variants, except for potassium sorbate, exhibited antimicrobial activity against *P. aeruginosa* American Type Culture Collection (ATCC) 27853. The results are listed in Table 1.

**Figure 1:**
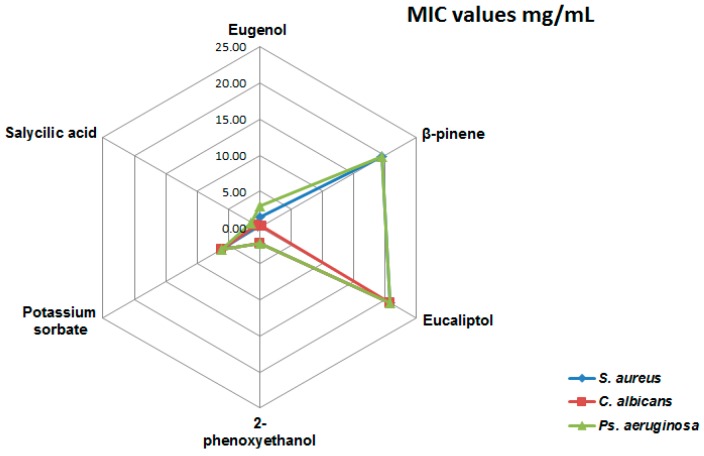
Minimal inhibitory concentration (MIC) values for the tested compounds (mg/mL).

In the present study, the minimal inhibitory concentration (MIC) values (mg/mL) of eugenol, eucalyptol, β-pinene, conventional preservatives, i.e., 2-phenoxyethanol, potassium sorbate, and salicylic acid, an antiacne active component, were under 5 mg/mL for all tested strains (Figure 1).

### 2.2. Fractional Inhibitory Concentration (FIC)/Fractional Inhibitory Concentration Index (FICI) of Natural Compounds: Salicylic Acid and Natural Compounds—Conservation Systems Mixtures

Mun et al. 2014 [19] proposed an interpretation model for the calculated FICI, i.e., ≤0.5, synergism of action; 0.5–0.75, partial synergism; >0.75–1, additive effect; >1, without effect, and >4, antagonistic effect. The tested mixtures harbored FICI values in the range of 0.5–0.75, demonstrating a partial synergism, except for the FICI calculated for Mixture 2, against the *C. albicans* strain, with a value of 1.23, suggesting a lack of interaction between the mixture’s components (Table 2).

### 2.3. Flow Cytometry Assessment of the Possible Mechanisms of Action of the Natural Components and Antimicrobial Mixtures

**Figure 2:**
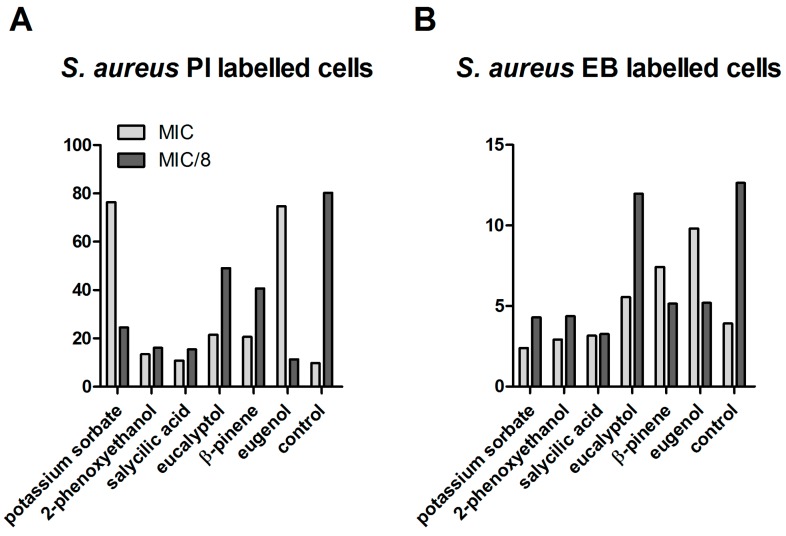
Median of fluorescence intensity (MFI) for the analytical variants tested on *S. aureus* strainATCC 6538. (**A**) MFI for *S. aureus* propidium iodide (PI)-labeled cells; (**B**) MFI for *S. aureus* ethidium bromide (EB)-labeled cells.

The membrane disruption potential, as well as efflux pumps inhibitor (EPI) activity by essential oils (EOs) was previously reported as a possible mechanism of their antimicrobial action [20,21]. Engel and Martins et al. 2013 proposed a correlation between the fluorescence intensity (FI) of the ethidium bromide (EB)-labeled cells with the ratio net influx of EB/EB extracellular elimination through the efflux pump activity ratio [22,23]. The median of fluorescence intensity (MFI) measurements revealed different aspects registered for the two tested concentrations and the tested microbial strains. In the case of *S. aureus*, at MIC, a drastic increase of the propidium iodide (PI) signal was associated with increased membrane permeability; thus, higher susceptibility to other physical or chemical antimicrobial agents was induced by eugenol and potassium sorbate. Eugenol, β-pinene, and eucalyptol at MIC values also exhibited an inhibitory effect on the efflux pumps, as revealed by the increased EB signal. In exchange, at MIC/8 concentrations, the permeability of bacterial cells was decreased by all of the tested compositions, and the majority of the tested compounds activated the efflux pumps activity, as revealed by the decreased EB fluorescence signal (Figure 2).

**Figure 3:**
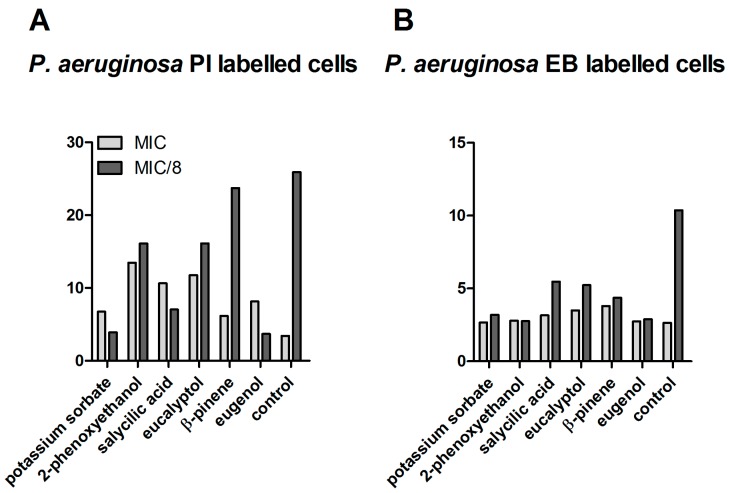
MFI for the analytical variants tested on *P. aeruginosa* strain ATCC 27853. (**A**) MFI for *P.aeruginosa* PI-labeled cells; (**B**) MFI for *P.aeruginosa* EB-labeled cells.

In the case of *P. aeruginosa*, at MIC values, a variable increase of the PI signal associated with increased membrane permeability was induced by all tested combinations, and particularly for eucalyptol, eugenol, 2-phenoxyethanol, and salycillic acid. Similar to *S. aureus*, at sub-inhibitory concentrations corresponding to MIC/8 concentrations, a significant efflux pumps inhibitory activity was registered for all tested compositions (Figure 3).

**Figure 4:**
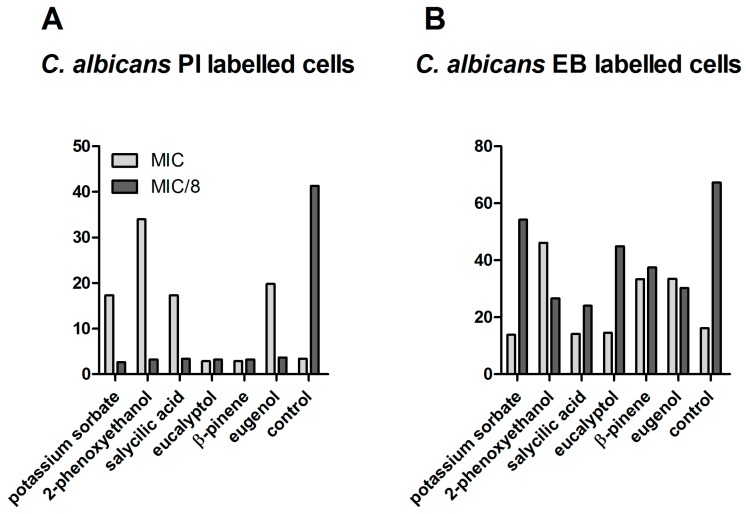
MFI for the analytical variants tested on *C. albicans* strain ATCC 10231. (**A**) MFI for *C. albicans* PI-labeled cells; (**B**) MFI for *C. albicans* EB-labeled cells.

In the case of *C. albicans* at MIC values, an increased membrane permeability and thus a higher susceptibility to other physical or chemical antimicrobial agents was induced only by eugenol, β-pinene, and the two preservative agents, whereas, at MIC/8 concentrations, the permeability of bacterial cells was drastically decreased by all of the tested compositions. A dose-dependent effect upon the activity of efflux pumps was also obtained for the fungal strain, similar to bacterial strains (i.e., inhibition at high concentrations and activation at sub-inhibitory concentrations) (Figure 4).

Taken together, these results demonstrate that, at sub-inhibitory concentrations, the tested compounds activate the efflux pums activity, preventing the accumulation of toxic compounds inside microbial cells and could be responsible for the occurrence of co-resistance to other antimicrobial substances. Therefore, the MIC values of the natural compounds that are intended to be introduced in antimicrobial formulations should be carefully determined in order to prevent the activation of efflux pumps implicated in the emergence of multiple resistance mechanisms.

**Figure 5:**
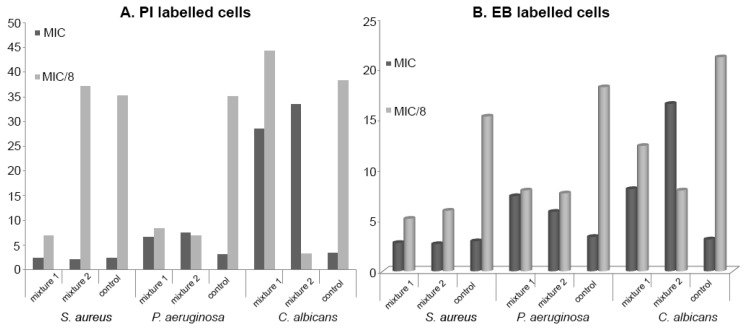
MFI for the analytical variants tested on the specified strains. (**A**) PI labeles cells; (**B**) EB labeled cells.

Concerning the flow cytometry analysis of the antimicrobial mixtures, a significant cellular permeabilization of *P. aeruginosa* and *S. aureus* cells was induced by both Mixture 1 and Mixture 2 at MIC/8 concentrations, while, in the case of *C. albicans*, only Mixture 1 exhibited this effect at MIC/8. Similar to individual tested components, the obtained mixtures exhibited a general efflux pumps activating effect at MIC/8 in the case of all tested strains (as seen in Figure 5).

### 2.4. Effectiveness of the Conservation System by the Challenge Test

Formal methods used to assess the effectiveness of various conservation systems and the similarities and differences between them were analyzed by several authors [24,25]. For this study, the challenge test was used. The topical formula conservation system meets Criterion A specified in the current European Pharmacopoeia edition (Table 3) [26].

Taken together, the obtained results revealed the eugenol/β-pinene/2-phenoxyethanol/potassium sorbate as the most potent antimicrobial combination, acting on at least two different microbial targets, i.e., cellular wall permeability and efflux pumps activity. In addition to their antimicrobial activity, the natural compounds included in the proposed formulation are also known to exhibit other anti-acne activities, such as free radical scavenging, anti-lipase [27], anti-inflammatory [28,29], and keratolytic [30].

## 3. Materials and Methods

### 3.1. Antibacterial Activity Assays

#### 3.1.1. Qualitative Screening of the Antimicrobial Activity of the Plant Compounds

The antimicrobial qualitative screening was carried out against standardized microbial strains recommended for the European Pharmacopoeia (8th ed.) microbiological quality assessment for topical drug formulations, i.e., *Staphylococcus aureus* ATCC 6538, *Pseudomonas aeruginosa* ATCC 27853, and *Candida albicans* ATCC 10231. The bacterial suspensions were obtained from cultures of 18–24 h grown on solid medium and adjusted to a density of 1.5 × 10^8^ CFU/mL, corresponding to the 0.5 McFarland standard. The tested mixtures/compounds have been diluted in DMSO (1:1) to facilitate the diffusion of the non-polar components in a culture medium and spotted on the previously seeded culture media in volumes of 10 µL. The interpretation of the growth inhibition diameters was performed using the Singh et al. algorithm, i.e., <8 mm—no antimicrobial activity, 9–14 mm—sensitive, 15–19 mm—very sensitive, and >20 mm—extremely sensitive [31].

#### 3.1.2. Quantitative Assay of Antimicrobial Activity Using the Liquid Medium Microdilution Method

Quantitative analysis was performed by the serial microdilution technique. Binary dilutions were obtained in a liquid medium (Müller-Hinton broth) distributed in 96 multiwell plates and standardized 0.5 McFarland microbial suspensions. Positive controls were untreated microbial cultures, and negative controls were represented by sterile culture medium with the specified dilutions for the compounds. The scheme of serial dilutions for the compounds is shown in Table 4. The plates were incubated at 24 h at 37 °C. Minimal inhibitory concentrations (MICs) were assessed by spectrophotometric reading of the optical density.

### 3.2. Determination of Fractional Inhibitory Concentration Index (FICI) for Eugenol/β-Pinene/Salicylic Acid (Mixture 1) and Eugenol/β-Pinene/Conservation System (Mixture 2)

The fractional inhibitory concentrations (FIC) and FICI (Table 5) were calculated based on the MIC results, using the following formula: FIC_mix individual components_ = MIC_mix_/MIC_mix individual components_; FICI**_mix/tested strain_** = $\sum_{n=1}^{4}\mathrm{FIC}\;$and FICI _mix_$\;=\sum_{i=1}^{j}{\mathrm{FICI}}_{\mathrm{mix}\;\mathrm{per}\;\mathrm{tested}\;\mathrm{strain}}*0.5$, where n is the number of the mixture components, and j is the number of calculated FICI.

### 3.3. Flow Cytometry Assessment of the Mechanism of Action for the Natural Components and Formulations

The flow cytometry assay was carried out in order to evaluate the possible mechanism of action of the tested compounds, as well as of their mixtures with the conventional preserving system salicylic acid—Mixture 1—or with 2-phenoxyethanol/potassium sorbate—Mixture 2. The tested concentrations for the flow cytometry assay were MIC and MIC/8. The antimicrobial combinations were included in a cream base formulation.

The fluorochromes used for the cell staining were propidium iodide (PI 10 µg/mL) and ethidium bromide (EB 5 µg/mL), allowing the investigation of the cellular coatings integrity (PI) and the efflux pumps activity (EB). The cells grown in the presence of the tested compounds at the above-mentioned concentrations were centrifuged at 12,000 rpm for 3 min, washed twice, re-suspended in PBS (adjusting, if necessary, the optical density OD_620_ of the suspended cells to 0.01–0.04 mau, corresponding to an inoculum under 10^7^ CFU/mL), stained, and incubated for 5 min at 4 °C in dark. Positive (heat-inactivated cells at 100 °C for 1 h) and negative (viable cells) controls for membrane permeabilization were used. After incubation, the samples were analyzed with a FACS Calibur instrument equipped with a 488 nm Argon laser, using filters—a 670 nm long pass filter for samples stained with PI and a 585 ± 42 nm band pass filter for the samples stained with EB. Forward scatter (FSC) vs. side scatter (SSC) and fluorescence measurements were taken, and the backgating procedure was used, excluding all nonfluorescent particles. Typical photomultiplier tube voltage parameters were as follows: SSC 550 V for bacteria and 450 V for fungal cells (log scale), a 670 nm long pass filter (log scale) and a 585 ± 42 nm band pass filter 550 V (log scale). A total of 10,000 events were collected in all runs.

### 3.4. Developed Formulation

An oil-in-water emulsion type formulation basis containing the tested antimicrobial combinations was prepared (Table 6). All components were of pharmaceutical or cosmetic grade, obtained from commercial sources (IQL), and the selection was based on their commercial availability and wide usage. The components of the oil phase were heated to melt and disperse, and the temperature was then maintained at 70 ± 2 °C for 90 min. The water phase components were heated to 70 ± 2 °C. The water phase was streamed into the oil phase with light mixing and then with sweep agitation, the mixture was cooled to 40 ± 2°C. After adding the preservatives and the thermolabile constituents, the mixture was homogenized with a rotor-stator homogenizer. The obtained emulsion was then cooled to 25 ± 2 °C under gentle agitation. Laboratory samples were obtained using a homogenizer IKA^®^ RET control/electronic contact thermometer 1 IKA^®^ ETS-D5. Thermodynamic stability of the emulsion was tested by cyclic temperature variation using the accelerated aging test method (40 °C for 48 h; 45 °C for 48 h) and the stability in the centrifuge field (3000 rpm for 30 min, at 25 °C). A homogeneous, thermodynamically stable emulsion with a pH of 5.5 and a viscosity of 3700–4000 mPa.s (40 S1) was obtained.

### 3.5. Microbiological Quality Control of the Obtained Formulation—Challenge Test

An efficacy test is a standard procedure that involves the artificial contamination of an obtained formulation with a predefined number of bacteria and fungi (10^5^−10^6^ viable cells mL^−1^ or g^−1^ in the cosmetic product), and periodic samples are taken at fixed time intervals to count the number of viable microorganisms present in the cosmetic formulation during the test. The used microorganisms include bacterial strains *S. aureus*, *P. aeruginosa*, and *Escherichia coli*, and fungi *C. albicans* and *Aspergillus niger* standardized strains. According to the European Pharmacopoeia, a topical preparation is well preserved if the number of microorganisms sampled per gram of the tested product is reduced by factors of 10^3^ (Criterion A) and 10^2^ (Criterion B) within two days of the challenge test, without cell proliferation in 7–28 days; for fungi, reduction growth factors of 10^2^ (Criterion A) and 10^1^ (Criterion B) within 14 days of the test are required [26].

## 4. Conclusions

The complex algorithm proposed in this study allowed the in vitro selection of natural active compounds for optimized combinations with antimicrobial activity, with applications in cosmetic and pharmaceutical industries. The possible mechanisms of action revealed by flow cytometry were cellular permeabilization and the inhibition of efflux pump activity induced by concentrations corresponding to sub-MIC values. The most active antimicrobial combination represented by eugenol/β-pinene/2-phenoxyethanol/potassium sorbate was included in a cream base formulation alongside salicylic acid as a well known antiacne compound.

## Figures and Tables

**Table 1:** Qualitative screening of the antimicrobial activity results; EA—Extremely active substances (>20 mm growth inhibition zone), A—active substances (15–18 mm), M—Moderate antimicrobial activity (9–14 mm), I—no antimicrobial activity (<8 mm).

Strain Analytical Standard	*S. aureus* ATCC 6538	*P. aeruginosa* ATCC 27853	*C. albicans* ATCC 10231
eugenol	EA	M	EA
β-pinene	A	M	EA
eucalyptol	M	M	EA
salicylic acid	EA	A	EA
2-phenoxietanol	A	EA	EA
potassium-sorbate	M	I	I

**Table 2:** Fractional inhibitory concentration index (FICI) values calculated for the tested strains/mixture.

Microbial Strains	FICI _Mixture 1_	FICI _Mixture 2_
*S. aureus*	0.572330839	0.520134776
*C. albicans*	0.626	1.23
*Ps. aeruginosa*	0.653571678	0.730516725

**Table 3:** Challenge test results.

Microbial strains	Inoculum Concentration	Logarithmic Reduction of Microbial Growth in the Obtained Formulation				
		T_0_	2 Days	7 Days	14 Days	28 Days
*S. aureus*	8.15	5.69	5.69	ga	nt	ga
*P. aeruginosa*	8.23	5.38	5.38	ga	nt	ga
*C. albicans*	8.04	4.39	nt	nt	4.39	ga
*A. brasiliensis*	7.78	5.3	nt	nt	5.3	ga

ga—growth absence; nt—not tested.

**Table 4:** The range of binary serial dilutions.

Binary Serial Dilutions	EO Fraction as Analytical Standard (µL/ mL)	Potassium Sorbate (mg/mL)	2-Phenoxyethanol (mg/mL)
C1	45.000	6.000	2.130
C2	22.500	3.000	1.065
C3	11.250	1.500	0.533
C4	5.625	0.750	0.266
C5	2.813	0.375	0.133
C6	1.406	0.188	0.067
C7	0.703	0.094	0.033
C8	0.352	0.047	0.017
C9	0.176	0.023	0.008
C10	0.088	0.012	0.004
C11	0.044	0.006	0.002
C12	0.022	0.003	0.001
C13	0.011	0.001	0.001
C14	0.005	0.001	0.001

**Table 5:** Receipt of tested formulations for the calculation of FIC/FICI.

Tested Mixtures	Percentage Concentration for the Components (%)				
	Eugenol	β-Pinene	Salicylic Acid	2-Phenoxyethanol	Potassium Sorbate
Mixture 1	0.7	3.17	0.17	-	-
Mixture 2	0.7	3.17	-	0.37	0.094

**Table 6:** Formulation composition.

No.	Formulation Composition	%
1	Water	ad. 100
2	Polyglyceryl-3-methylglucose distearate	3.20
3	*Olea Europaea* Fruit Oil	3.00
4	*Macadamia integrifolia* seed oil	3.00
5	Isopropyl palmitate	3.00
6	Squalane	3.00
7	Glycerin	3.00
8	Isononyl isononanoate	3.00
9	Meadowfoam estolide	3.00
10	Caprylic/capric triglyceride	2.00
11	Glyceryl stearate	2.00
12	Stearyl alcohol	1.00
13	Water and potassium sorbate and phenoxyethanol	0.50
14	Eugenol/β-pinene	1
15	Xanthan gum	0.50
16	Bisabolol	0.10
17	EDTA	0.05

## Abbreviations

MIC	minimal inhibitory concentration
FIC	fractional inhibitory concentration
FICI	fractional inhibitory concentration index
